# Analytic Optimization of Cantilevers for Photoacoustic Gas Sensor with Capacitive Transduction

**DOI:** 10.3390/s21041489

**Published:** 2021-02-21

**Authors:** Wioletta Trzpil, Nicolas Maurin, Roman Rousseau, Diba Ayache, Aurore Vicet, Michael Bahriz

**Affiliations:** IES, University Montpellier, CNRS, 34095 Montpellier, France; wioletta.trzpil@umontpellier.fr (W.T.); nicolas.maurin@ies.univ-montp2.fr (N.M.); roman.rousseau@ies.univ-montp2.fr (R.R.); diba.ayache@ies.univ-montp2.fr (D.A.); aurore.vicet@umontpellier.fr (A.V.)

**Keywords:** MEMS, gas sensor, photoacoustics, cantilever, capacitive detection, analytic model

## Abstract

We propose a new concept of photoacoustic gas sensing based on capacitive transduction which allows full integration while conserving the required characteristics of the sensor. For the sensor’s performance optimization, trial and error method is not feasible due to economic and time constrains. Therefore, we focus on a theoretical optimization of the sensor reinforced by computational methods implemented in a Python programming environment. We present an analytic model to optimize the geometry of a cantilever used as a capacitive transducer in photoacoustic spectroscopy. We describe all the physical parameters which have to be considered for this optimization (photoacoustic force, damping, mechanical susceptibility, capacitive transduction, etc.). These parameters are characterized by opposite trends. They are studied and compared to obtain geometric values for which the signal output and signal-to-noise ratio are maximized.

## 1. Introduction

The market for gas sensors was estimated to be 2.23 billion USD in 2020 and is expected to reach 4.49 billion USD in 2028 [1]. The growing interest in gas sensors is driven by various field of applications, e.g., medicine [2], air quality [3], food processing [4], or security and defense [5], that address legislative (e.g., EU’s air quality directives), National Ambient Air Quality Standards) and/or individual needs. Sensors commonly used in the market, according to the highest percentage contribution into the gas sensor market income, are electrochemical, semiconductor, and infrared sensors [1]. Electrochemical sensor principles are based on creation of an electrical signal after reaction with a target gas. Semiconductor sensors are made of heated metal oxides which in the presence of the gas change their resistivity. Infrared gas sensors are based on electromagnetic signal conversion into electrical signal [6]. Characteristics of these sensors are presented in Table 1 [7].

Gas sensors for real-life applications [7], e.g., air quality, toxic gasses, medicine, food processing, are required to be selective (perfectly distinguish one species among others), sensitive (able to detect few particles per million in volume (ppmv)), reliable (stable, suffer from small drift), and compact. Infrared gas sensors, like the ones based on tunable diode laser spectroscopy (TDLS), can perfectly discriminate the spectral signature of a gas species among others, thus providing an excellent selectivity, combined with a high sensitivity (sub-ppb detection) [8] (Table 1). The main drawbacks of infrared detection are: lack of absorption line in infrared spectrum for some gasses, poor selectivity for gasses with absorption line at the same wavelength, and lack of compactness.

Photoacoustic spectroscopy, an evolution of TDLS, permits reducing the size of the gas sensor while maintaining equivalent performances. In TDLS the detected signal is proportional to the length of the optical path while in photoacoustic spectroscopy, it is related to the laser emitted power, which allows keeping a high sensitivity even in a compact gas cells.

In photoacoustic spectroscopy, a modulated laser emitting at a wavelength corresponding to the absorption line of a targeted gas species is focused into a gas chamber. The measurement is performed by detecting the acoustic pressure generated by the local warming induced by molecular relaxation following optical absorption. The local temperature rise is a result of non-radiative vibrational–translational (V-T) relaxation processes occurring between excited molecules. At atmospheric pressure, the laser emission linewidth (∼MHz) is much smaller than the gas linewidth (∼GHz), which gives a perfect selectivity to this method.

The acoustic wave can be measured using a microphone [11] or a mechanical resonator such as a tuning fork [12]. The use of a mechanical resonator with high quality factor (around 10,000 for a quartz tuning fork (QTF)) improves the signal-to-noise (SNR) ratio and avoids the use of a resonant acoustic chamber.

Commercial QTF allows reaching very good sensing performances in Quartz Enhanced Photoacoustic Spectroscopy (QEPAS) [13] even if they were developed for the electronics market, and not for sensing purposes. As a consequence, the QTF is not optimized for photoacoustic spectroscopy and its potential integration in a compact system is limited compared to other mechanical resonators based on silicon materials. Silicon would offer several advantages such as its technological maturity, its design flexibility and its lower production costs. However, its best advantage lies in the feasibility of integration in complex CMOS electronics [14,15]. Recent progress in laser sources integration on silicon [16] makes it possible to consider fully integrated compact sensors. For these reasons, silicon-based micro-resonator seems to be the best choice for the future development of very compact gas sensors integrated on the same chip with electronics, a laser, and a mechanical resonator.

We study here the realization of a silicon-based micro resonator sensor, a cantilever, dedicated to photoacoustic sensing. This sensor, specifically designed for acoustic sensing purposes, would be an efficient transducer for sound wave detection.

The most common transduction methods in silicon-based micro-electromechanical systems (MEMS) are based on capacitive, piezoresistive, and piezoelectric effects. The capacitive transduction mechanism constitutes a more convenient method than piezoelectric [17] or piezoresistive [18] detection. It avoids any material deposition or implantation on the mechanical resonator, which may reduce the quality factor and make the fabrication process more complex. Capacitive detection employed in MEMS technology allows reaching high sensitivity. For example, the capacitive accuracy for accelerometers or position sensors is about a few ppm of their nominal capacitance [19], leading to a sub-femto-farrad resolution [20]. To improve a capacitive signal, it is advantageous to increase the capacitor surface which leads to a rise in viscous damping and abbreviates the devices performances. Undoubtedly, for parameters characterized by opposite trends, an optimization based on a theoretical model would be the first step towards sensor performance improvement.

**Figure 1:**
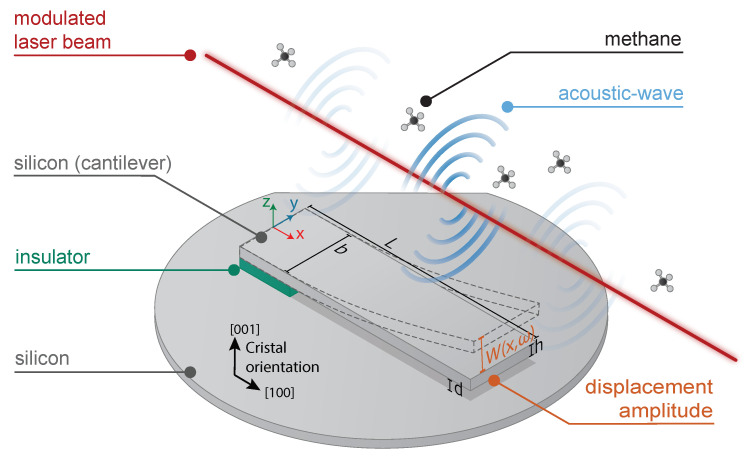
Sensing scheme of a silicon cantilever-based sensor for photoacoustic gas detection with capacitive transduction mechanisms.

The working principle of a gas sensor based on photoacoustic spectroscopy using a cantilever as a capacitive transducer is schematically presented in Figure 1. The acoustic pressure generated by laser light absorption applies a force on the cantilever and sets it in motion. To maximize the displacement, the acoustic wave is generated at the resonance frequency of the cantilever via laser wavelength modulation. The silicon cantilever is electrically insulated from the back silicon, forming a capacitor. One of the electrodes of the capacitor is the cantilever itself. The displacements of the cantilever cause the capacitance variations. Depending on the excitation frequency, the capacitance variations can be converted into a current or a voltage signal.

Performing solely a trial and error method for the sensor’s performance optimization is not feasible due to economic and time constraints. Therefore, a computational method is the most reasonable choice. The sensing scheme imposes multi-physics problems in different domains and can be divided in four parts: (1) acoustic force, (2) damping mechanisms, (3) mechanical displacement, and (4) output signal. Many of these problems are not directly coupled and others are characterized by opposite trends in terms of geometry optimization. The main novelty in our approach is a simultaneous multi-physics optimization. This optimization aims to determine the geometrical parameters of the cantilever (length *L*, width *b*, thickness *h*, gap *d* (Figure 1)) and its resonance frequency, which would maximize the output electrical signal and the signal-to-noise ratio. For this, the cantilever has to be sized to maximize its displacement under acoustic wave exposition while exhibiting a strong capacitance variation.

The paper is divided as follows:

(1) The acoustic force part describes the generation of the photoacoustic wave and its interaction with the cantilever. As in TDLS, the acoustic wave is obtained by wavelength modulation technique at the pulsation ω [21]. The photoacoustic effect is related to heat production rate, depending on the non-radiative relaxation time of the target gas. The non-radiative relaxation strongly relies on the molecular species, the gas concentration, temperature, pressure, and the gas mixture. To obtain realistic values, our numerical estimation is performed on one specific gas: $CH_{4}$ diluted in $N_{2}$. However, the model is compatible with any gas mixture once the relaxation time is known.
(2) The damping mechanism part describes the following mechanisms of losses: viscous, thermoelastic, support, and acoustic damping.
(3) The mechanical part describes how the cantilever is set in motion by the acoustic wave, taking into account its susceptibility. The displacement amplitude of the cantilever is described by W(x,ω) presented in Figure 1. Further terms describing the cantilever displacement refer to the fundamental vibration mode presented in Figure 1.
(4) The output signal part represents the energy conversion from mechanical motion to an electrical signal. It gives the relation between the cantilever deflection W(x,ω) and the electrical signal output $V_{out}\left(\omega \right)$.
(5) The SNR part illustrates the thermal noise $W_{noise}\left(x,\omega \right)$.

## 2. Acoustic Force

**Figure 2:**
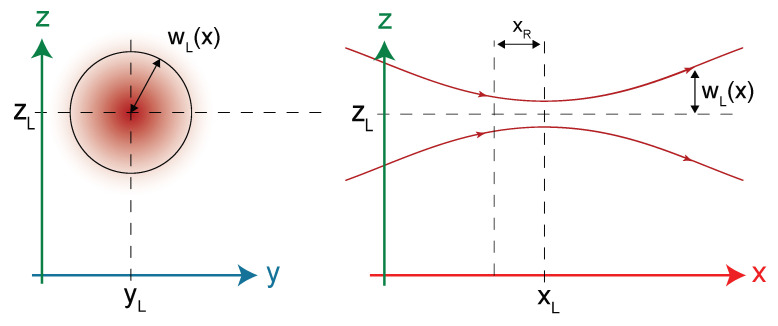
Gaussian beam profile and its position on the axis in relation to the cantilever microbeam. $w_{L}\left(x_{L}\right)$ = 100 μm, $\lambda_{L}$ = 1.65 μm, $x_{L}$ = 0.725L, $y_{L}$ = 0, $z_{L}$ = 150 mm.

The purpose of this section is to study the cantilever dimensions (length *L*, width *b*, thickness *h*) and its resonance frequency in order to maximize the acoustic force. This part evaluates the photoacoustic pressure generation and the photoacoustic force applied to the cantilever. The source of the photoacoustic wave generation lies in periodic gas absorption induced by a modulated laser beam. This method is called wavelength modulation spectroscopy [21]. We consider a Gaussian laser beam propagating along the x-axis at an altitude $z=z_{L}$ and centered with respect to y-axis at $y=y_{L}$ (Figure 2).

The distribution of the light intensity I(x,y,z) is related to the laser power $P_{L}$:

(1) $$I\left(x,y,z\right)=P_{L}g\left(x,y,z\right)$$

$$g\left(x,y,z\right)=\frac{2}{\pi w_{L}{\left(x\right)}^{2}}\exp\left(-2\frac{{\left(z-z_{L}\right)}^{2}+{\left(y-y_{L}\right)}^{2}}{w_{L}{\left(x\right)}^{2}}\right)$$

where g(x,y,z) is a normalized Gaussian profile and $w_{L}\left(x\right)=w_{L}\left(x_{L}\right)\sqrt{\left(1+\frac{{\left(x-x_{L}\right)}^{2}}{x_{R}^{2}}\right)}$ is the laser radius which depends on the Rayleigh length $x_{R}=\frac{\pi w_{L}\left(x_{L}\right)}{\lambda_{L}}$, with $\lambda_{L}$ the laser emission wavelength.

The theoretical model used to describe the pressure and force of the acoustic wave generated by molecular absorption is based on the model developed by Petra et al. [22]. However, our model takes into account the variation of the laser beam radius $w_{L}\left(x\right)$ along the optical axis (x-axis) and the effects of the gas relaxation time constant. The assumptions used in the model are:

1. The wavelength modulation is performed without modulation of the laser power.
2. Sommerfeld radiation conditions: no reflection from any walls of a gas cell and photoacoustic energy fading at infinity.
3. The photoacoustic pressure is unaltered by the presence of the cantilever.
4. The laser beam radius is smaller than the distance between the cantilever and the optical axis.

To fulfill the third assumption, the acoustic wave wavelength $\lambda_{a}$ must be at least one order of magnitude larger than the thickness and width of the cantilever: $\lambda_{a}∼3.5$ cm at ν = 10 kHz ($\lambda_{a}=\frac{c_{s}}{\nu }$, where $c_{s}$ is the speed of sound).

The absorption of the modulated light causes periodic heat changes and subsequently an acoustic wave. The heat production rate is given by:

(2) $$H\left(x,y,z,t\right)=\frac{C_{f}\left(\omega \right)g\left(x,y,z\right)}{\sqrt{1+{\left(\omega \tau \right)}^{2}}}e^{i(\omega t-\arctan\left(\omega \tau \right))}$$

where ω is the laser modulation frequency, τ is the target gas relaxation time $C_{f}\left(\omega \right)$ is the effective absorption coefficient. The absorption and transmission line shapes can be ideally described with a Lorentzian line shape function. The laser emission wavelength scan the absorption line and is modulated around the central wavelength $\lambda_{c}$. The modulated wavelength can be expressed as: $\lambda \left(t\right)=\lambda_{c}+\lambda_{amp}sin\left(\omega t\right)$, where $\lambda_{amp}$ is the modulation amplitude. When the laser wavelength is modulated the power remains constant and equal to $P_{L}$, we can write $C_{f}\left(\omega \right)=0.50\alpha \left(\omega \right)P_{L}$, where α(ω) is the absorption coefficient of the gas. The 0.5 factor is obtained by expansion of the absorption function in Fourier series. We consider only the first Fourier component ($a_{1}=0.5$) for the 1f detection method. The second Fourier components would result in a coefficient of 0.35 ($a_{2}=0.35$) [22].

The photoacoustic wave generation is related to the heat production due to the light absorption. The expression of photoacoustic pressure P(x,y,z) is given by the wave equation:

(3) $$\frac{\partial^{2}P\left(x,y,z,t\right)}{\partial t^{2}}-c_{s}^{2}\Delta P\left(x,y,z,t\right)=\left(\gamma -1\right)\frac{\partial H(x,y,z,t)}{\partial t}$$

where $c_{s}=347.276$ m/s is the sound velocity in air and $\gamma =\frac{C_{p}}{C_{v}}$ the adiabatic gas coefficient or heat capacity ratio equal to the fraction ratio between heat capacities at constant pressure and volume.

Equation (3) is an inhomogeneous equation with time. By substituting $P\left(x,y,z,t\right)=p\left(x,y,z\right)e^{i\omega t}$ and $H\left(x,y,z,t\right)=h\left(x,y,z\right)e^{i\omega t}$ and imposing Sommerfeld radiation boundary conditions, Petra et al. [22] showed that the pressure equation takes the following form:

(4) $$p\left(x,y,z\right)=-\frac{\pi A}{2c_{s}^{2}k_{s}^{2}}\left(Y_{0}\left(k_{s}r\right)+iJ_{0}\left(k_{s}r\right)\right)\int_{0}^{+\infty }uJ_{0}\left(u\right)\exp\left(\frac{-2u^{2}}{k_{s}^{2}w_{L}{\left(x\right)}^{2}}\right)\;du$$

where $J_{0},Y_{0}$ are the zero-order Bessel functions of the first and the second kind, respectively. $A=-\left(\gamma -1\right)\omega H\left(x,y,z\right)\frac{2}{\pi w_{L}{\left(x\right)}^{2}}$ represents the amplitude of photoacoustic pressure, $k_{s}=\omega /c_{s}$ is the wave number, and $r=\sqrt{{\left(z-z_{L}\right)}^{2}+{\left(y-y_{L}\right)}^{2}}$ is the distance between the laser beam and the cantilever.

The photoacoustic force $F_{PA}$ applied on the cantilever is defined as the difference of pressure between the top and bottom surfaces of the cantilever.

(5) $$F_{PA}=\int_{0}^{L}\int_{-b/2}^{b/2}\left(p(x,y,z)-p(x,y,z-h)\right)\phi_{n}\left(x\right)\;dx\;dy$$

$\phi_{n}\left(x\right)$ describes the one-dimension shape of the cantilever mechanical mode *n*. It gives the cantilever deflection and can be found analytically by solving an eigenvalue problem of the Euler–Bernoulli equation. The mode shape for a clamped-free cantilever is given by [23]:

(6) $$\phi_{n}\left(x\right)=\cosh\left(\alpha_{n}\frac{x}{L}\right)-\cos\left(\alpha_{n}\frac{x}{L}\right)-\frac{\sinh\left(\alpha_{n}\right)-\sin\left(\alpha_{n}\right)}{\cosh\left(\alpha_{n}\right)+\cos\left(\alpha_{n}\right)}\left(\sinh\left(\alpha_{n}\frac{x}{L}\right)-\sin\left(\alpha_{n}\frac{x}{L}\right)\right)$$

The acoustic force acting on the cantilever is frequency-modulated at the wavelength modulation frequency of the laser source. For a first harmonic detection (1f detection) it is adjusted to the cantilever mode frequency. In our model the cantilever vibrates at its fundamental mode-first harmonic n=1 which corresponds to a mode constant $\alpha_{1}=1.875$.

### Results and Discussion

**Figure A1:**
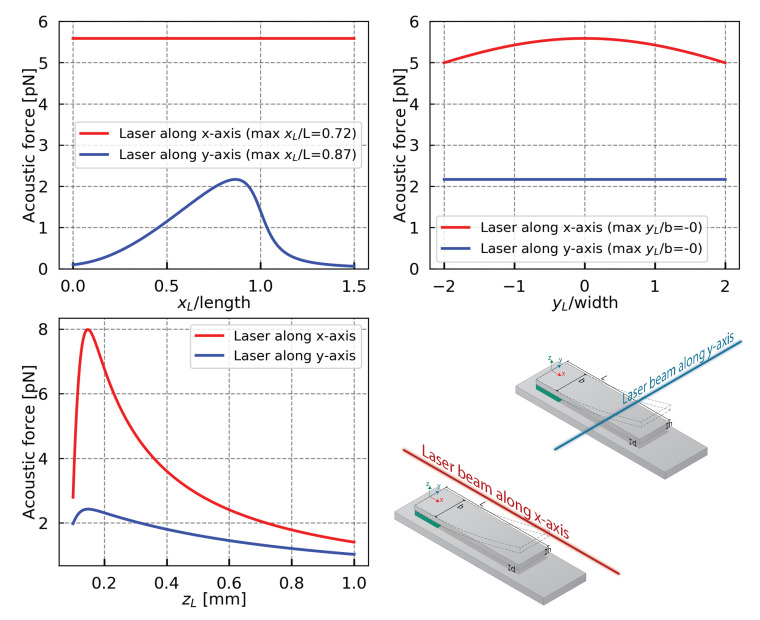
Amplitude of the photoacoustic force applied on a cantilever as a function of the laser beam position for two configurations: red—laser beam along the x-axis; blue—laser beam along the y-axis. Where $f_{n}=11$ kHz, b=50μm, h=100μm, and L=3.5 mm, respectively are the resonance frequency, the width, the thickness, and the length of the cantilever.

The parameters used in the simulation are detailed in Table 2. We chose a laser emitting at 1.65μm to target a strong methane $CH_{4}$ absorption line. Based on our numerical simulation presented in Appendix A.1, Figure A1 illustrates how $x_{L}$ and $y_{L}$ coordinates maximize the acoustic force, while $z_{L}=250\;\mu$m conserves the assumption that laser light does not interfere with the cantilever.

Due to the thermal relaxation time, the modulation frequency strongly affects the heat production rate (Equation (2)) and subsequently the acoustic force. Indeed, to allow the molecules to thermalize efficiently, the laser modulation needs to be lower than the molecules relaxation time.

Each molecule exhibits a different relaxation time. To maximize the photoacoustic force, the optimisation needs to be made with respect to one type of gas. We chose $CH_{4}$ diluted in nitrogen $N_{2}$ for which the relaxation time is equal to 11.5 μs [24]. However, the relaxation time between the molecules might differ by several orders of magnitude.

**Figure 3:**
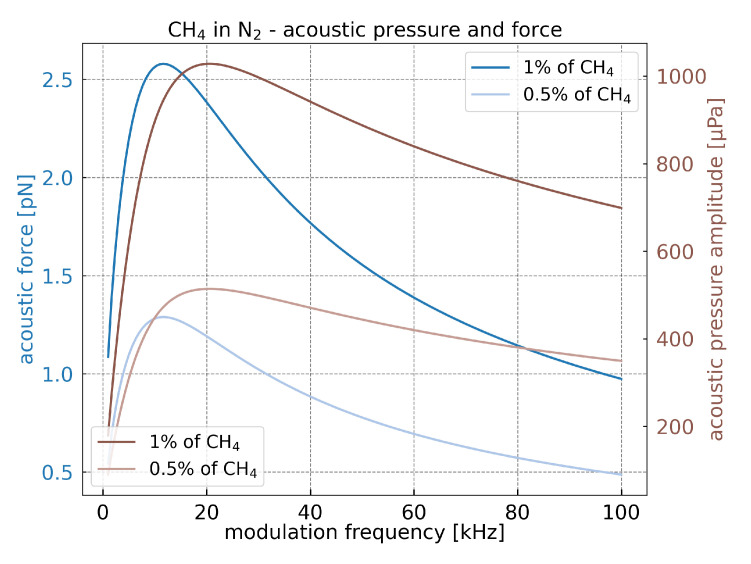
Acoustic force and acoustic pressure dependency on the modulation frequency for diluted $CH_{4}$ at 1% and 0.5% in nitrogen. Cantilever width b=25μm and thickness h=100μm.

Figure 3 presents the acoustic pressure and force for $CH_{4}$ diluted in $N_{2}$, 1% and 0.5%, respectively. Only the acoustic force depends on cantilever geometry. To maintain a fixed frequency, the cantilever length is adjusted with the following equation:

(7) $$f_{n}=\frac{\omega_{n}}{2\pi }=\frac{\alpha_{n}^{2}}{2\pi \sqrt{12}}\frac{h}{L^{2}}\sqrt{\frac{E}{\rho_{b}}}$$

where $f_{n}$ is the resonance frequency of a clamped-free cantilever, $\rho_{b}$ = 2330 kg/m${}^{3}$ is the silicon density and E=130 GPa is Young’s modulus for silicon in [100] direction [25].

The values of the acoustic force and pressure clearly depend on the modulation frequency as it is presented in Figure 3. For each concentration, they increase with the frequency until reaching a maximum around 20 kHz for the acoustic pressure and around 11 kHz for the acoustic force. This maximum is related to $CH_{4}$ relaxation time value. The maximum shift to lower frequency between the acoustic pressure and the acoustic force is due to the cantilever length which appears only in the acoustic force, Equation (5). According to Equation (7), the length of the cantilever is longer for lower frequencies. Therefore, the surface exposed to the acoustic pressure is larger, which subsequently increases the acoustic force at low frequencies.

The maximum value of the acoustic force is at 11 kHz. To maximize the force applied on the cantilever, this frequency is used in the following numerical simulations of the cantilever geometry (width *b*, thickness *h*, and length *L*). However, the model is adaptable to any frequency with respect to the assumptions.

**Figure 4:**
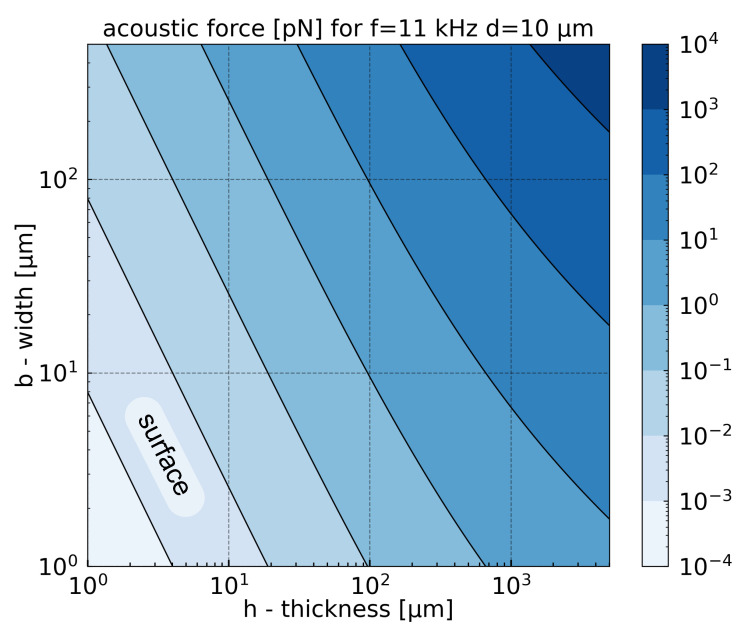
Acoustic force for 1% of $CH_{4}$ in $N_{2}$ as a function of width *b* and thickness *h* of the cantilever. For different thickness, the length is adjusted to maintain constant frequency: 11 kHz. This frequency was chosen to maximize acoustic force. The area with the weakest acoustic force corresponds to cantilevers with the smallest surface.

Figure 4 represents the total photoacoustic force applied on cantilever for different cantilever geometries. It shows two general trends. Firstly, the photoacoustic force increases with the width *b* and the thickness *h*. Indeed, the surface enlargement increases the energy collection from the acoustic wave. Secondly, the thickness increment increases the pressure difference between the top and bottom sides of the cantilever, which enhances the acoustic force. For a fixed cantilever frequency, the increase of the thickness causes the length increment and enlarges the total surface (Equation (7)). The results presented in Figure 4 would change while using different gases, different volume mixing ratios, or different frequencies (Figure 3). Nevertheless, the general trend would remain constant.

The results presented in the following sections are further taken into the calculation to get the optimized geometry of the cantilever with regard to electrical signals and signal-to-noise ratio.

## 3. Damping Mechanism—Quality Factor

The quality factor *Q* is a dimensionless number which describes the energy losses in the system. It can be expressed as the ratio between the energy stored in a cycle of vibration $E_{stored}$ and the energy dissipated in a cycle of vibration $E_{dissipated}$

(8) $$Q=2\pi \frac{E_{stored}}{E_{dissipated}}$$

There are two main mechanisms where the cantilever can lose energy: through internal energy dissipation, like thermoelastic losses, and external dissipation, like viscous damping, support losses or acoustic losses. The total quality factor consists of quality factors originating from different losses and can be calculated using the following equation:

(9) $$\frac{1}{Q_{total}}=\frac{1}{Q_{viscous}}+\frac{1}{Q_{thermo}}+\frac{1}{Q_{support}}+\frac{1}{Q_{acoustic}}$$

### 3.1. Thermoelastic Losses

Thermoelastic damping (TED) is a loss mechanism due to the irreversible heat flow in vibrating structures. A temperature gradient occurs between regions under tension (where the temperature drops) and regions under compression (where the temperature rises).

We use an analytical model proposed by Lifshitz [26,27], where the thermoelastic quality factor is given by:

(10) $$Q_{thermo}=\frac{C_{p}}{E\alpha_{T}^{2}T}{\left(\frac{6}{\xi^{2}}-\frac{6}{\xi^{3}}\left(\frac{\sinh(\xi )+\sin(\xi )}{\cosh(\xi )+\cos(\xi )}\right)\right)}^{-1}$$

ω, $C_{p}$, $\alpha_{T}$, *T*, *E* are the pulsation, specific heat capacity, linear thermal expansion coefficient, temperature, Silicon Young’s modulus, respectively. $\xi =h\sqrt{\frac{\omega \rho_{b}C_{p}}{2K}}$ represents a dimensionless number where *K* is the thermal conductivity. The values of all these parameters can be found in Table 3. The maximum of thermoelastic damping [26] occurs for ξ=2.225. This value corresponds to a transition frequency $f_{t}=\frac{\pi }{2}\frac{K}{\rho_{b}C_{p}h^{2}}$. For a cantilever frequency $f_{n}$ lower than the transition frequency $f_{t}$ ($f_{n}<f_{t}$), the beam is permanently in thermal equilibrium. In this case the vibration is called isothermal. On the other hand, when $f_{n}>f_{t}$ the cantilever frequency is higher than the transition frequency, the beam does not have enough time to thermally equilibrate and this vibration is called adiabatic. In both cases, the energy dissipation is low. However, the $Q_{thermo}$ quality factor is higher in isothermal than in adiabatic regime [28]. In case of constant-frequency regime, one needs to calculate the thickness that gives the maximal damping. Based on the $f_{t}$ expression, the isothermal zone corresponds to the thin cantilever thickness and the adiabatic zone to the large thickness. For $f_{n}=11$ kHz, the maximal thermoelastic damping, i.e., the lowest $Q_{thermo}=12,500$, corresponds to a cantilever with thickness h=90μm. Therefore, for frequency of 11 kHz, thermoelastic damping is not a limiting factor.

### 3.2. Acoustic Losses

Acoustic losses refer to losses caused by a vibrating structure being a source of acoustic wave radiation. A good approximation of these losses can be expressed with an analytical model for cantilever with elliptical cross-section [29,30,31]. In this approach the quality factor related to acoustic losses is given by the following equation:

(11) $$Q_{acoustic}=\frac{256}{\pi }\frac{\rho_{b}}{\rho_{f}}\frac{1}{{\left(k_{s}b\right)}^{3}}\frac{h\int_{0}^{L}\phi_{n}^{2}\left(x\right)\;dx}{\int_{\varphi =0}^{\pi }sin^{3}\varphi {\left|\int_{0}^{L}\phi_{n}\left(x\right)\exp\left(-ik_{s}xcos\left(\varphi \right)\right)\;dx\right|}^{2}\;d\varphi }$$

where $\rho_{f}$ is a fluid density, $k_{s}=\omega /c_{s}$ the acoustic wave number and $c_{s}$ is the speed of sound. Numerical calculations using Equation (11) show that the losses due to acoustic radiation become important when the cantilever length is comparable to the acoustic wavelength $\lambda_{a}$. It is less significant at low frequencies. Moreover, acoustic losses increase quickly as the width increases and the thickness decreases (for constant-frequency regime). For instance, for b=5000μm and h=1μm $Q_{acoustic}≃605$.

### 3.3. Support Losses

The cantilever presented in Figure 1 is held by a support. During the cantilever movement a part of the energy is dissipated into the support. This dissipation is described by the support quality factor. An analytical solution for support losses in case of a clamped-free cantilever was proposed by Hao [32] and takes the following form:

(12) $$Q_{support}=\left(\frac{0.24(1-\nu )}{(1+\nu )\Psi }\right)\frac{1}{{\left(\frac{\alpha_{n}}{\pi }\chi_{n}\right)}^{2}}{\left(\frac{L}{h}\right)}^{3}$$

where $\nu ,\alpha_{n},\chi_{n}$ is the Poisson’s ratio, a mode constant, and a mode shape factor, respectively. For the clamped-free cantilever fundamental mode n=1 and $\alpha_{1}=1.875$, the mode shape factor $\chi_{1}=\frac{\sin\left(\alpha_{1}\right)-\sinh\left(\alpha_{1}\right)}{\cos\left(\alpha_{1}\right)+\cosh\left(\alpha_{1}\right)}$ and Ψ=0.336. It can be seen from Equation (12) that the energy dissipation from the support is inversely proportional to ${\left(L/h\right)}^{3}$. If we look at a fixed frequency, without considering the length of the cantilever, then the quality factor of the support is $Q_{support}\propto \frac{1}{\sqrt{\omega_{n}^{3}h^{3}}}$.

### 3.4. Viscous Damping

Viscous damping originates from the fluid resistance. It is considered to be the most significant damping mechanism in MEMS operating in ambient conditions.

**Figure 5:**
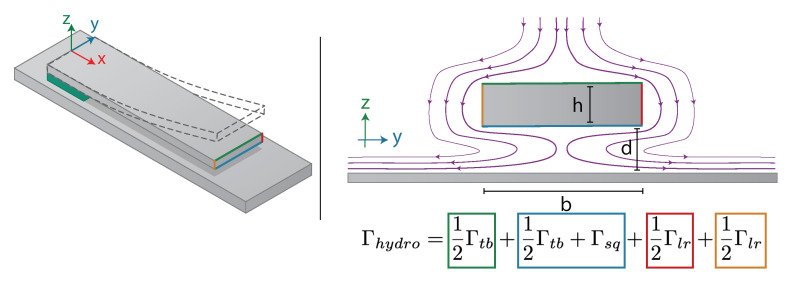
Scheme of streamlines acting on the cross-section sidewalls of the cantilever oscillating in its first mode of vibration with corresponding hydrodynamic functions. $\Gamma_{tb}$ is used to describe the forces applied at the top and the bottom of the cantilever, $\Gamma_{lr}$ relates to the left and right sides of the cantilever, while $\Gamma_{sq}$ is a hydrodynamic force originating from squeeze film effect.

During the beam movement in fluid, an additional force related to the medium appears. The quality factor due to viscous damping can be analytically expressed using a normalized time-independent function called hydrodynamic function $\Gamma_{hydro}$:

(13) $$Q_{viscous}=\frac{\frac{4\rho_{b}h}{\pi \rho_{f}b}+\Gamma_{hydro}^{R}\left(\omega \right)}{\Gamma_{hydro}^{I}\left(\omega \right)}$$

where $\rho_{b}$, $\rho_{f}$, $\Gamma_{hydro}^{R}$, $\Gamma_{hydro}^{I}$ are the density of the beam, the density of the fluid, and the real and imaginary parts of the hydrodynamic function, respectively. The total hydrodynamic function originates from the linearized Navier–Stokes equation. Thus, it can be represented as a linear combination of hydrodynamic functions originating from each sidewall of the beam cross-section [33]. Pictorially, it is presented in Figure 5, while mathematically it is expressed as:

(14) $$\Gamma_{hydro}=\frac{1}{2}\Gamma_{tb}+\frac{1}{2}\Gamma_{tb}+\Gamma_{sq}+\frac{1}{2}\Gamma_{lr}+\frac{1}{2}\Gamma_{lr}$$

where $\Gamma_{tb},\Gamma_{sq},\Gamma_{lr}$ are hydrodynamic functions originating from the top and bottom side of the cantilever, squeeze film, and the left and right side of the cantilever, respectively.

#### 3.4.1. Viscous Damping on the Top and the Bottom

$\Gamma_{tb}$ describes the viscous damping on the front and the back of the cantilever.

Sader [34] used the exact analytic solution for a circular-cross section cantilever. Then he used a multiplicative correction function $\Omega_{sader}$ in order to provide a more precise result in case of infinitely thin rectangular beams. Correction function $\Omega_{sader}$ depends on the Reynolds number and therefore on the width and frequency of the cantilever. The expression of $\Gamma_{tb}$ is given in Equation (15) and the $\Omega_{sader}$ expression in [34].

(15) $$\Gamma_{tb}\left(\omega \right)=\left(1+\frac{4iK_{1}\left(-i\sqrt{iR_{e}}\right)}{\sqrt{iR_{e}}K_{0}\left(-i\sqrt{iR_{e}}\right)}\right)\Omega_{sader}\left(\omega \right)$$

$K_{0}$, $K_{1}$ are modified Bessel functions of the second kind, $Re=\frac{\rho_{f}\omega b^{2}}{4\mu_{f}}$ is the Reynolds number, $\rho_{f}$ is the density of the fluid, and $\mu_{f}$ is the dynamic viscosity.

#### 3.4.2. Viscous Damping on the Left and on the Right

The theoretical approach of $\Gamma_{lr}$ can be found in [33] and takes the following form:

(16) $$\Gamma_{lr}\left(\omega \right)=\frac{2\sqrt{2}h}{\pi b\sqrt{R_{e}}}\left(1+i\right)$$

As explained in [35], this expression neglects edge and thickness effects, but remains a sufficient approximation in our configuration since it has been tested and compared to experimental results in [33].

#### 3.4.3. Viscous Damping Due to the Squeeze Film Effect

On the cantilever sidewall, where the gas is trapped between substrate and cantilever, there exists an additional counter reactive force originating from squeeze film action. A mathematical description of squeeze film was given by Bao et al. [36]:

(17) $$\Gamma_{sq}\left(\omega \right)=\frac{-4P_{a}}{\pi db\rho_{f}\omega^{2}}\left(f_{e}\left(\sigma \right)-if_{d}\left(\sigma \right)\right)$$

where $P_{a}$, *d* are the surrounding pressure and air gap shown in Figure 5, σ is a squeeze number [36] given by the following equation:

(18) $$\sigma =\frac{12\mu_{f}\omega L^{2}}{P_{a}d^{2}}$$

and $f_{e}\left(\sigma \right)$ and $f_{d}\left(\sigma \right)$ are functions introduced by Langlois [37] with the following form:

(19) $$\left(\begin{array}{ccc} f_{e}\left(\sigma \right) & = & 1-\sqrt{\frac{2}{\sigma }}\frac{\sinh\left(\sqrt{\frac{\sigma }{2}}\right)+\sin\left(\sqrt{\frac{\sigma }{2}}\right)}{\cosh\left(\sqrt{\frac{\sigma }{2}}\right)+\cos\left(\sqrt{\frac{\sigma }{2}}\right)}\\ f_{d}\left(\sigma \right) & = & \sqrt{\frac{2}{\sigma }}\frac{\sinh\left(\sqrt{\frac{\sigma }{2}}\right)-\sin\left(\sqrt{\frac{\sigma }{2}}\right)}{\cosh\left(\sqrt{\frac{\sigma }{2}}\right)-\cos\left(\sqrt{\frac{\sigma }{2}}\right)} \end{array}\right)$$

### 3.5. Results and Discussion

**Figure 6:**
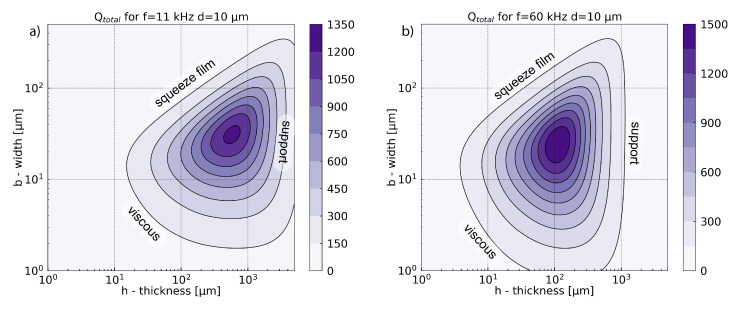
Total quality factor as a function of width and thickness for a cantilever of fundamental resonance frequencies equal to 11 kHz (**a**) and 60 kHz (**b**), for a gap between support and cantilever equal to d=10μm.

Despite the lack of a general trend for the quality factor optimization in terms of all losses, it is possible to find the optimal value of the quality factor in terms of geometry for a given frequency. This optimum is presented in Figure 6. It takes into account all damping mechanisms presented in the previous subsections. The values of parameters used in this numerical simulation are presented in Table 3. Simulations have been realized at 11 kHz where the acoustic force is maximal, and for comparison at 60 kHz. An optimum has been found for laser modulation frequency at 11 kHz. For other physical mechanisms like damping mechanism, other optimums in modulation frequency can be expected. Simulation at higher frequency illustrates the evolution of these physical parameters with the frequency. As it will be seen in Section 6, although the different frequency dependency of physical mechanisms, the optimum frequency for the gas sensor is the same as the one for the acoustic force, here 11 kHz. A complete frequency study is presented in Appendix A.4. For the present simulations, a gap value of d=10μm has been chosen as a good compromise between fabrication constraint and sensor performances. The extensive study of the gap is presented in Section 7. Simulations show that the maximum value is slightly larger at high frequencies, and the most significant effect is the shift of the optimum to the lowest thickness when the frequency increases. As we will detail below, this effect is due to the losses of the mechanical supports.

In the figure, we identified the areas which correspond to the main limiting mechanisms. As it was shown $Q_{support}\propto \frac{1}{\sqrt{\omega_{n}^{3}h^{3}}}$, therefore the limitation for the quality factor with high thickness originates from the damping of the support. The term in $\omega^{3}$ in this equation explains the shift of the optimum to the lowest thickness, when the frequency increases.

The effect of the squeeze film damping appears for the largest width when the gas is trapped under the cantilever. For the smallest width, where the inertial forces are smaller than the viscous forces, the total quality factor is limited by the viscous damping. This area corresponds to the lowest Reynolds number.

**Figure A2:**
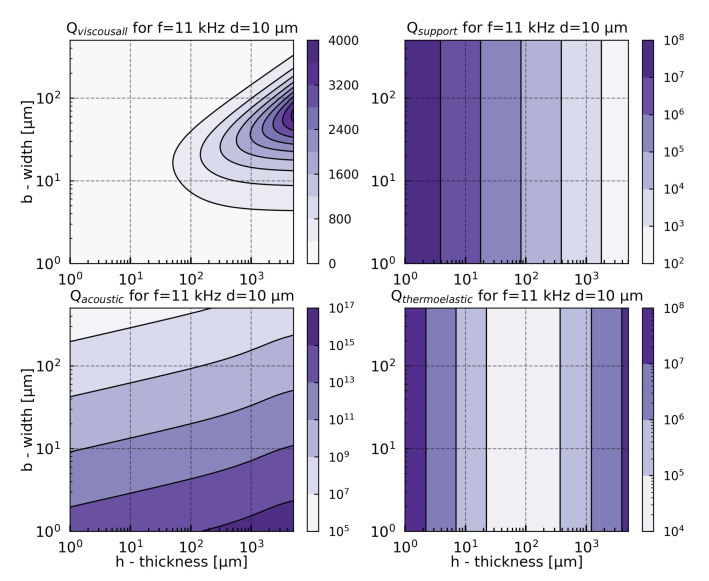
Quality factors for viscous damping with squeeze film (top-left panel), support damping (top-right panel), acoustic damping (bottom left panel), and thermoelastic damping (bottom-right panel) as a function of width and thickness of a cantilever. Cantilever’s fundamental resonance frequency: 11 kHz, gap between support and cantilever: d=10μm.

**Figure A3:**
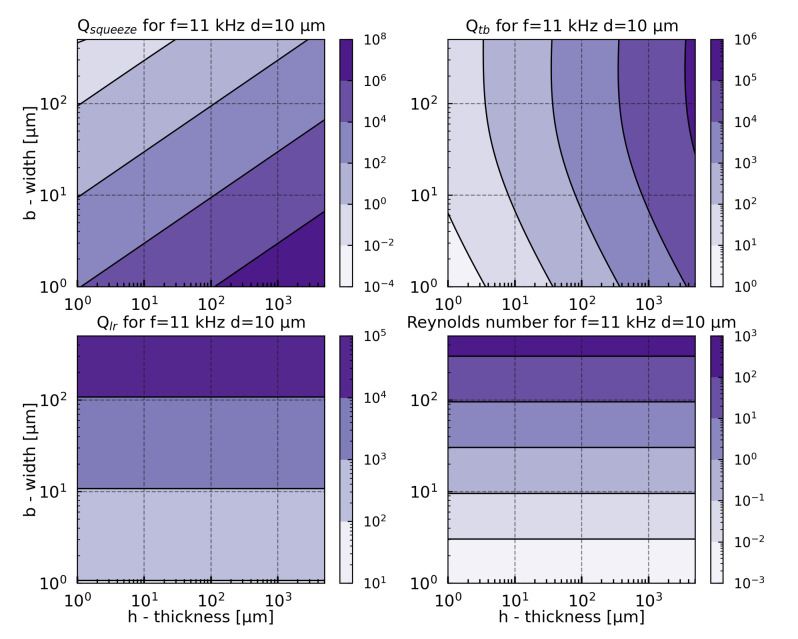
Quality factors for squeeze film (top-left panel), viscous damping originating from the top and bottom side of a cantilever (top-right panel), viscous damping originating from left and right side of a cantilever (bottom left panel) and Reynolds number (bottom-right panel) as a function of width and thickness of a cantilever. Cantilever’s fundamental resonance frequency: 11 kHz, gap between support and cantilever: d=10μm.

In this model, neither thermoelastic nor acoustic damping are limiting factors. For all geometries and frequencies, the two associated quality factors are at least one order of magnitude higher than the other damping mechanisms. For more detail on the individual limits of each quality factor, the reader can refer to Appendix A.2, Figure A2 and Figure A3.

## 4. Displacement

The deflection $W_{n}\left(x,\omega \right)$ at the position *x* of the n-th mode of the beam under a photoacoustic driving force $F_{PA}\left(\omega \right)$ is given by:

(20) $$W_{n}\left(x,\omega \right)=\chi_{n}\left(\omega \right)F_{PA}\left(\omega \right)\phi_{n}\left(x\right)=w_{n}\left(\omega \right)\phi_{n}\left(x\right)$$

where $\chi_{n}\left(\omega \right)$, $F_{PA}\left(\omega \right)$, $\phi_{n}\left(x\right)$, $w_{n}\left(\omega \right)$ are the mechanical susceptibility, photoacoustic driving force, mode shape function normalized with $max(\phi_{m}\left(x\right))=1$, and the maximal displacement, respectively. For the fundamental mode $w_{n}\left(\omega \right)$ denotes the displacement amplitude at the extremity of the beam. The susceptibility represents the frequency-dependent response of the cantilever under an external force and can be expressed as:

$$\chi_{n}\left(\omega \right)=\frac{1}{m_{n}\left(\omega_{n}^{2}-\omega^{2}\right)+i\left(\frac{\omega_{n}\omega m_{n}}{Q_{total}}\right)}$$

where $Q_{total},m_{n}$ are the total mechanical quality factor and the effective mass, respectively. The effective mass represents the part of structure actually involved in the movement.

The structure is subjected to two opposite forces: the photoacoustic force $F_{PA}$ which is periodic and drives the beam into motion and the resistance caused by damping of the structure. The damping is given by the total quality factor $Q_{total}$. Both forces are presented in previous sections. The effective mass is described by:

(21) $$m_{n}=\rho_{b}hb\int_{0}^{L}\phi_{n}^{2}\left(x\right)\;dx$$

It is related to the resistance of the resonator for motion changes. Consequently, it decreases the susceptibility and amplitude displacement of the resonator.

### Results and Discussion

**Figure 7:**
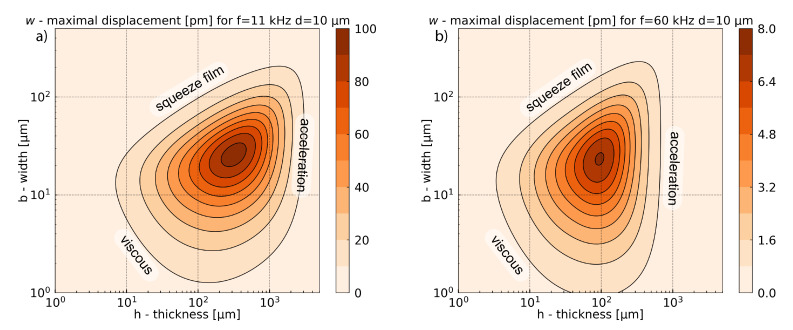
Total displacement versus width and thickness for a cantilever with fundamental resonance frequency equal to 11 kHz (**a**) and 60 kHz (**b**) for a gap between support and cantilever equal to d=10μm.

Figure 7 has been calculated with the mathematical expression of the previous section (Equation (4)). In this section, some approximations will be proposed to explain the shape of the graph.

The mechanical displacement $w_{n}\left(\omega_{n}\right)$ presented in Figure 7 is the product between the photoacoustic force and the mechanical susceptibility $\chi_{n}$. At the resonance frequency $2\pi f_{n}=\omega_{n}$, the susceptibility can be approximated as $\chi_{n}=Q/\left(m_{n}\omega_{n}^{2}\right)$ and the displacement as $w_{n}\left(\omega_{n}\right)=Q_{total}F_{PA}/(\omega_{n}^{2}m_{n}$).

For the fundamental mode of the cantilever, we can write the acoustic force as $F_{PA}≃0.39bL\Delta p\left(b,h\right)$, where Δp(b,h) is the pressure difference between the top and the back of the cantilever. The function Δp(b,h) increases with the thickness *h* and in this approximation remains quite constant for various widths *b*. The effective mass of the cantilever fundamental mode is $m_{n}≃0.25\rho_{b}hbL$ (i.e., 25% of the total mass). The displacement can then be approximated by:

(22) $$w_{n}\left(\omega_{n}\right)=\frac{Q_{total}F_{PA}}{\omega_{n}^{2}m_{n}}≃1.56\frac{Q_{total}}{\omega_{n}^{2}}\frac{\Delta p(b,h)}{\rho_{b}h}$$

The simulations show that the fraction $\frac{\Delta p(b,h)}{\rho_{b}h}$ remains quite constant for different widths and is inversely proportional to the cantilever thickness: $\frac{\Delta p(b,h)}{\rho_{b}h}\propto \frac{1}{h}$. Due to its homogeneity, this term is called “acceleration” in Figure 7. It is reducing the maximal displacement when the thickness increases. This region corresponds to weak acoustic force or/and heavy effective masses. Counterintuitively, the simplified Equation (22) shows that increasing the cantilever surface to collect more photoacoustic energy increases the effective mass, resulting in constant mechanical displacement. Indeed, a simplification by the surface bL appears between the term of the acoustic force and the effective mass.

The other limitations come from the viscous damping introduced by the $Q_{total}$ term, and are similar to those shown in the Figure 6.

Figure 7 shows a large difference in displacement amplitude between a modulation frequency of 11 kHz and 60 kHz. Despite the improvement of quality factor with increasing frequency, the acoustic force significantly drops at high frequency (Figure 3) and the susceptibility, as it is inversely proportional to $\omega_{n}^{2}$. The results then show that photoacoustic force and susceptibility gain more importance with the change of frequency.

## 5. Electrical Part

This section focuses on maximizing the conversion between mechanical deflection and electrical signal.

The nominal capacitance $C_{0}=Lb\varepsilon_{0}\varepsilon_{r}/d$ is the capacitance value without any displacement, where $\varepsilon_{r},\varepsilon_{0}$ are the relative permittivity of the media (equal to unity in air) and vacuum, respectively. For the different geometries considered, $C_{0}$ may take values between $10^{-4}$ and 100pF. The expression of nominal capacitance indicates that the change of the distance between two electrodes will cause a capacitance variation.

The dynamic capacitance caused by deflection of the cantilever is given by [38]:

(23) $$C\left(t\right)=\int_{0}^{L}\frac{b\varepsilon_{0}\varepsilon_{r}}{d+W_{n}\left(x,\omega \right)}\;dx\exp\left(i\omega t\right)$$

**Figure 8:**
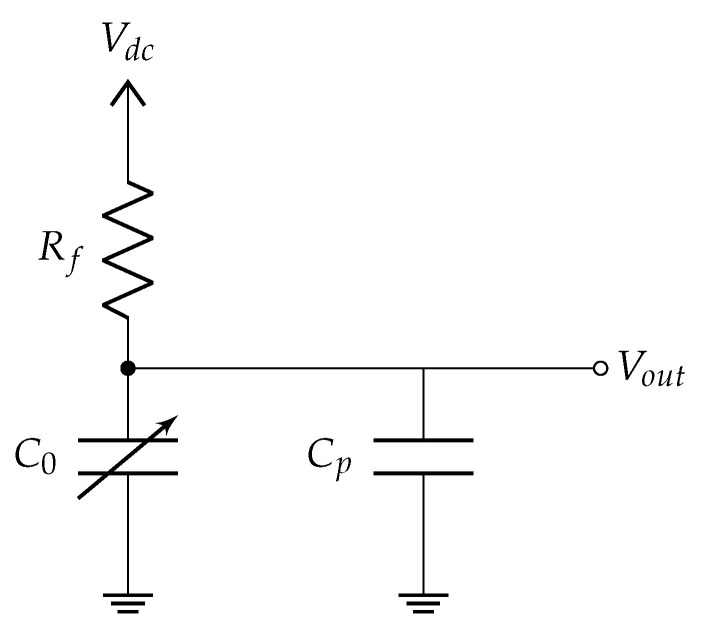
Sensor conditioning circuit, where: $C_{0},C_{p},R_{f},V_{dc},V_{out}$ are the capacitance of the cantilever, parasitic capacitance, resistance, polarization voltage, and output voltage, respectively.

The model applies a method called DC bias sensing [39] (Chapter 5). Figure 8 presents the sensing scheme. In an electromechanical system, a polarization voltage $V_{dc}$ on the electrodes is required to generate an electrical signal related to the mechanical behavior of the moving electrode.

The application of the force generated by the photoacoustic effect sets the movable electrode in motion. This movement causes the changes of the capacitance from the maximal value $C_{max}$ to the minimal $C_{min}$. $C_{max}$ and $C_{min}$ correspond to the minimal and maximal distances between the cantilever and support, respectively.

The capacitance variation can take place at a constant charge or a constant voltage [40]. If the time constant $R_{f}C_{0}>>1/\omega_{n}$, the electric charge stored in the capacitor remains constant. $R_{f}≃100\;G\Omega -10\;T\Omega$ is the value of the resistor placed between the cantilever and the polarization voltage $V_{dc}$. In the constant charge regime, the voltage of the measured signal is given by $V_{out}\left(t\right)=C_{0}V_{dc}/C\left(t\right)$ while its amplitude is given by:

(24) $$V_{out}=V_{dc}\int_{0}^{L}\frac{W_{n}\left(x,\omega \right)}{Ld}\;dx$$

### Results and Discussion

**Figure 9:**
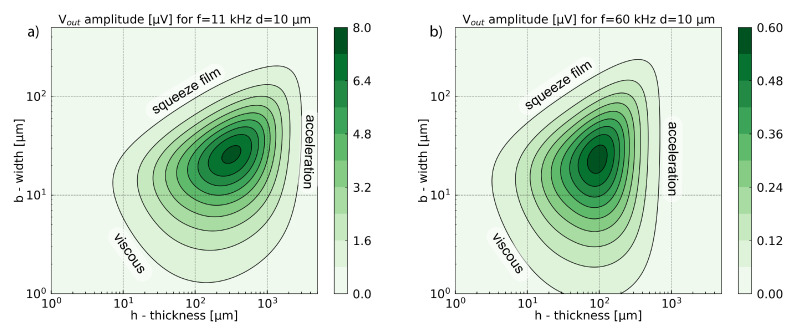
Amplitude of the output voltage versus width and thickness for a cantilever of fundamental resonance frequency equal to 11 kHz (**a**) and 60 kHz (**b**) for a gap between the support and the cantilever equal to d=10μm. The polarization voltage is $V_{dc}=1\;V$.

Figure 9 presents the results obtained for a bias $V_{dc}=1\;V$. The maximum values follow the tendencies given by the displacement. The values change with the gap *d* and frequency $f_{n}$. Indeed, according to Equation (22), Equation (24) can be simplified as follows:

(25) $$V_{out}≃0.39V_{dc}\frac{w_{n}\left(\omega \right)}{d}$$

Decreasing the gap *d* between the two electrodes should lead to an output signal amplitude increase. However, simultaneously it increases the squeeze film damping and reduces the cantilever displacement. The optimization of this parameter will be discussed in the last section.

Equation (25) indicates that the signal output does not depend on the area of the capacitor as it would be expected based on Equation (23). The output signal amplitude is given for an open circuit, without any read-out circuit which can modify the signal. In a complete system, the signal is attenuated by a parasitic capacitance $C_{p}$, which is the sum of the parasitic capacitance of the resonator itself and the one which comes from read-out circuit. The output signal attenuation can be estimated with the ratio $C_{0}/\left(C_{0}+C_{p}\right)$ [41].

## 6. Thermal Noise

**Figure A4:**
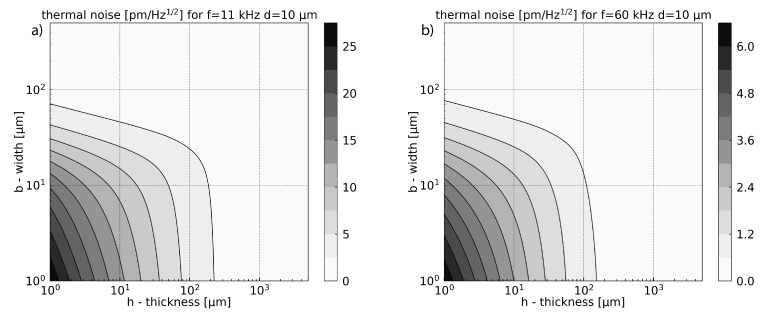
Thermal noise as a function of width and thickness of a cantilever. Cantilever’s fundamental resonance frequency: 11 kHz (**a**) and 60 kHz (**b**), gap between support and cantilever: d=10μm.

The sensor performance is limited by the unavoidable noise caused by the thermal fluctuations (Brownian movement) which set the resonator in motion. Therefore, it must be considered to construct a high-performance sensor. The maximum displacement of the cantilever caused by the Brownian noise $w_{noise}\left(\omega_{n}\right)$ [42] is given by the fluctuation-dissipation theorem:

(26) $$w_{noise}\left(\omega_{n}\right)=\sqrt{\frac{4k_{b}T\Delta fQ}{\omega_{n}^{3}m_{n}}}=\sqrt{4k_{b}T\Delta f}\sqrt{\frac{w_{n}}{\omega_{n}F_{PA}}}$$

where $k_{b}$, *T*, and Δf are the Boltzmann constant, cantilever temperature, and detection bandwidth, respectively. The plot for thermal noise as a function of cantilever geometry is presented in the Appendix A.3, Figure A4.

The signal-to-noise ratio at the resonance pulsation $\omega_{n}$ is given by:

(27) $$SNR=\frac{w_{n}\left(\omega_{n}\right)}{w_{noise}\left(\omega_{n}\right)}=\sqrt{\frac{w_{n}\omega_{n}F_{PA}}{4k_{b}T\Delta f}}$$

### Discussion about Signal-to-Noise Ratio

**Figure 10:**
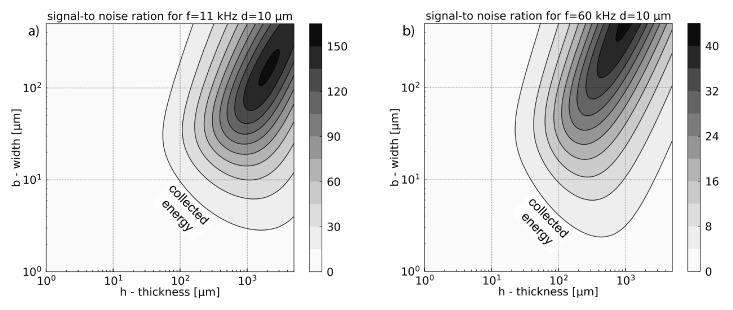
Signal-to noise ratio for a cantilever of fundamental resonance frequency equal to 11 kHz (**a**) and 60 kHz (**b**), a gap between support and cantilever equal to d=10μm.

The optimum for the SNR presented in Figure 10 does not match with the highest output signal amplitude presented in Figure 9. The highest output signal corresponds to the highest mechanical displacement $w_{n}$ (Equation (25)). The Brownian noise can be considered as a force acting on the cantilever. The optimal way to improve SNR is maximization of the photoacoustic force which comes down to increasing the surface area for photoacoustic pressure collection. Therefore, the optimum SNR is shifted to greater widths and thicknesses, where the acoustic force is greater (Figure 4), and the collected energy increases. As in the previous section, the increase of the surface collecting the photoacoustic energy will be limited to the larger widths by the squeeze film damping, and by the acceleration term for the larger thicknesses.

The second significant parameter in Equation (27) is the pulsation $\omega_{n}$. Unlike the previous result, $\omega_{n}$ reduces the difference between low and high frequencies. This is due to the fact that SNR is inversely proportional to the square root of frequency $SNR\propto \frac{1}{\sqrt{\omega_{n}}}$, while the amplitude of displacement $w_{n}\left(\omega_{n}\right)$ is inversely proportional to the frequency square power $w_{n}\left(\omega_{n}\right)\propto \frac{1}{\omega_{n}^{2}}$. For instance, the ratio between maximal value of SNR at 11 kHz and 60 kHz $\frac{max(SNR(11kHz))}{max(SNR(60kHz))}≃\frac{1}{22}\frac{max(w_{n}\left(11kHz\right))}{max(w_{n}\left(60kHz\right))}$ is around 22 times lower than the ratio of displacement. This indicates that for SNR the frequency term is less significant than in terms of displacement (voltage output).

## 7. Study of the Gap Effect

**Figure 11:**
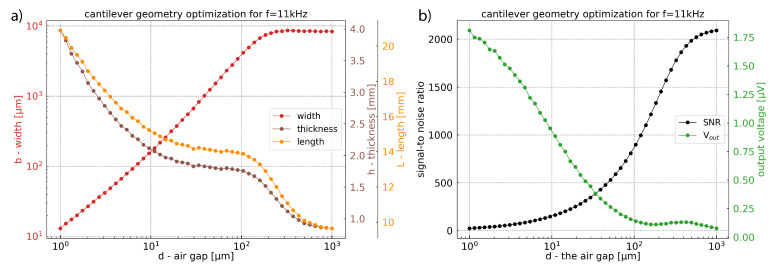
(**a**) Geometrical values giving the highest signal-to-noise ratio as a function of the gap *d* between support and cantilever. (**b**) Highest signal-to-noise ratio and its corresponding output amplitude signal as a function of the gap *d* between support and cantilever. For these simulations, the cantilever fundamental resonance frequency is equal to 11 kHz.

This section focuses on the effect of the distance between electrodes *d* (Figure 1) on the general sensor performance for a constant resonance frequency of 11 kHz. The analytic solutions previously presented (Equation (27)) were implemented in a Python programming environment to estimate an optimal value of signal-to-noise ratio for each value of the gap *d*. Subsequently, for each optimal SNR value, we get the geometrical parameters of the cantilever (width, length, thickness, Figure 11a) and their corresponding output voltages (Figure 11b).

When *d* increases, the signal-to-noise ratio increases as the displacement increases due to the decrease of squeeze film damping. At the same time, the signal output voltage decreases due to the increase of the distance between electrodes. This improvement on the SNR can also be explained by the optimized width, which increases with the gap *d* and which allows more energy collection. This increase in width is made possible by the decrease in the squeeze film damping for large gap *d*. Above d≃200μm the acoustic damping becomes dominant and some saturation appears on the width curve. The variations on the thickness curve are less important than on the width. The length curve follows the thickness rise to satisfy the constant frequency condition. As for the curve of the width, we can identify on the curves for length and thickness two different regimes that are probably due to the transition where acoustic damping becomes more important than squeeze film damping.

By taking into account the fabrication process issues, a cantilever with a gap *d* = 10 μm can be realized on a silicon-on-oxide (SOI) wafer. In this case, the SNR ratio will reach 150 and the amplitude of the output signal should reach 0.9 μV. Depending on the possibilities of the fabrication process, size of the final design, and required performance of the device (SNR), Figure 11 can be used as a reference to create a cantilever for optimal photoacoustic gas detection with capacitive transduction mechanisms.

## 8. Conclusions

The presented sensor combines multiple physical phenomena and, therefore, its optimization is not straightforward. In this paper, we discussed multiple parameters which affect the sensor performances and we analyzed their contributions. Our model takes into account some optimization for (1) the acoustic force; (2) the system damping; (3) the mechanical displacement under photoacoustic force considering damping mechanism; (4) the electrical signal under capacitive transduction mechanism, and (5) the signal-to-noise ratio. This model provides a method to retrieve the optimized cantilever geometry depending on the required size of the sensor and other restrictions which might be imposed by the fabrication process or the operating conditions of the sensor.

(1) Our model includes the gas relaxation time. We proposed a cantilever geometry optimized for photoacoustic gas sensor with a capacitive transduction mechanism in the context of $CH_{4}$ absorption with a concentration of 1%. For the same temperature and pressure conditions, only the amplitude of the photoacoustic force will change with the concentration. For this reason, the geometric parameters of the cantilever will remain the same for all the concentrations. For different gasses, only the optimal laser modulation frequency and the absolute acoustic pressure will change; the trends presented in other sections will remain the same. Based on our model, the cantilever optimal geometry can be recalculated for any other gas by taking into account the relaxation time and absorption coefficient. However, the optimal sensor should be created for one specific gas.

(2) The study of the different damping mechanisms show that viscous damping, and particularly squeeze film effect, is fundamental. The impact of squeeze film effect is visible in Figure 11 for *d* values up to 200μm. For *d* above this value, the acoustic damping becomes the limiting damping mechanism.

(3)–(5) To improve the SNR, one can increase the gap *d* and/or the surface collecting the photoacoustic energy. However, increasing the gap *d* between the two electrodes will decrease the output signal amplitude. Depending on the read-out circuit and the parasitic capacitance it is possible to obtain a high SNR while maintaining a sufficient output signal. For example, with a gap of *d* = 10 μm which can be realized on a silicon-on-oxide (SOI) wafer, the signal-to-noise ratio will reach 150 and the amplitude of the output signal will be around 0.9μV. For different values of the gap, one can use Figure 11.

Finally, despite a complex multiphysical problem, we have proposed a complete analytic model able to find the optimum geometric parameters of a cantilever for photoacoustic sensing with capacitive transduction. Beyond the simple optimization, this study is intended to provide all the tools allowing understanding of all the mechanisms of this complex problem. The variety of these physical mechanisms, often incompatible with each other during a finite element simulation, gives all its strength to our analytical approach to the problem. This paper demonstrates that a simple cantilever with capacitive transduction mechanism will not reach the same performance in terms of limit of detection as the best QEPAS technique or best standard photoacoustic technique using a microphone. However, besides being the optimization tool, this work is intended to be an educational tool allowing a mechanical resonator to be developed with more complex geometry and other transduction mechanisms. This study paves the way to develop new mechanical resonators for compact, integrated, and sensitive gas sensors.

## Figures and Tables

**Table 1:** Characteristics for various types of gas sensors based on [7].

Parameters	Electrochemical	Semiconductor	Infrared
sensitivity	g	e	e
stability	b	g	g
selectivity	g	p	e
compactnesss	p	e	b
cost	e	g	p
application	air purity [9]	Industrial applications and civil use [10]	(a) Remote air quality monitoring; (b) Gas leak detection systems; (c) High-end market applications. [10]

e—excellent, g—good, p—poor, b—bad.

**Table 2:** Parameters used to describe the laser source and the acoustic wave.

Parameter	Description	Value
$\lambda_{L}$	laser wavelength	1.65μm
$w_{L}\left(x_{L}\right)$	laser waist (experimental value)	100μm
$x_{L},y_{L},z_{L}$	laser beam waist radius position	0.725 L, 0, 250 mm
$P_{L}$	laser power	50 mW
$C_{gas}$	$CH_{4}$ concentration in $N_{2}$	1%
α	absorption coefficient	CH${}_{4}$0.38 m${}^{-1}$
τ	target gas relaxation time	CH${}_{4}$ 11.5 μs [24]
$c_{s}$	speed of sound	Air 347.276 m/s
γ	heat ratio capacity	Air 1.4

**Table 3:** Parameters used to describe the damping mechanism.

Parameter	Description	Value
$\rho_{b}$	mechanical resonator density	Si 2330 kg/m${}^{3}$
$\rho_{f}$	fluid density	Air 1.177 kg/m${}^{3}$
$\mu_{f}$	fluid dynamic viscosity	Air $1.85\times 10^{-5}$ kg/m/s
*d*	air gap	10 μm
$P_{a}$	pressure	101,325 Pa
T	temperature	300 K
*E*	Young’s modulus	Si${}_{<100>}130$ GPa
ν	Poisson’s ratio	Si${}_{<100>}$ 0.28
$\alpha_{T}$	thermal expansion coefficient	Si $2.6\times 10^{-6}$ 1/K
$C_{p}$	specific heat at constant pressure	Si 700 J/(kgK)
K	thermal conductivity	Si 90 W/m/K

**Figure A5:**
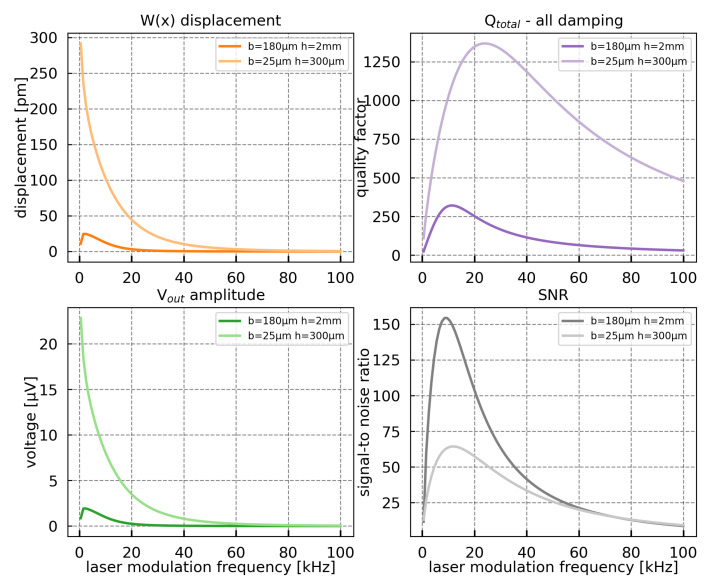
Frequency study for two geometries of cantilever with a distance between the electrodes d=10μm: geometry optimized for SNR (b=180μm, *h* = 2 mm) and geometry optimized for $V_{out}$ (b=25μm, *h* = 300 μm).

## Data Availability

Data and Python code developed for this article is available by contacting the correspoding author.

## Sample Availability

The Python code developed for this article is available by contacting the correspoding author.

## Abbreviations

The following abbreviations are used in this manuscript:

MEMS	micro-electromechanical systems
PA	photoacoustic
QEPAS	quartz enhanced photoacoustic spectroscopy
QTF	quartz tuning fork
SNR	signal-to-noise ratio
SOI	silicon-on-oxide
TDLS	tunable diode laser spectroscopy
V-T	vibrational–translational
